# Diet and Atrial Fibrillation: Does α‐Linolenic Acid, A Plant Derived Essential Fatty Acid, Have An Impact?

**DOI:** 10.1161/JAHA.112.000030

**Published:** 2013-02-22

**Authors:** Devera W. Kastner, David R. Van Wagoner

**Affiliations:** 1 Cleveland Clinic, Cleveland, OH (D.W.K., D.R.W.)

**Keywords:** Editorials, atrial fibrillation, diet, fatty acid

## Introduction

Many studies in the past 50 years have shown that dietary lipids can modulate the progression of coronary artery disease, heart failure, and cardiac arrhythmogenesis. Polyunsaturated fatty acids (PUFAs) are small biologically active lipid molecules that serve as a substrate for metabolic activity, cellular signals, and as precursors for signaling molecules such as prostaglandins, leukotrienes, etc. Small structural changes between PUFAs, especially with respect to chain length and the number and position of double bonds have a significant impact on their biological activities.^1^ PUFAs can modulate ion channel activity, cardiac myocyte contractility and Ca^2+^ cycling, and cell survival in response to ischemia or mechanical stress.

Epidemiologic evidence suggests that dietary consumption of fatty fish (salmon, sardines, and herring) lowers the risk of incident atrial fibrillation (AF).^2^ Fish oil contains a complex mix of ω3‐PUFA, ω6‐PUFA, ω9‐PUFA, and other lipids and antioxidant molecules. The most abundant long chain ω3‐PUFAs in fatty fish are eicosapentaenoic acid (EPA, 20 carbon) and docosahexaenoic acid (DHA, 22 carbon).^1^ In an attempt to identify which components of fish oil confer benefit in AF prevention, additional studies have sought to separately evaluate the impact of EPA and DHA on risk of AF. In 2 such studies, consumption of DHA, but not EPA was associated with lower risk of AF.^3–4^ It is thus plausible that dietary consumption of DHA has a meaningful impact on AF risk.

While fish are the most concentrated natural source of EPA and DHA, α‐linolenic acid (ALA) is the most abundant plant based ω3‐PUFA. Can dietary consumption of ALA, an abundant, sustainable and inexpensive ω3‐PUFA, lower the risk of AF in the same manner that consumption of fish and DHA appear to do? In this issue of the *JAHA*, Fretts and colleagues^5^ directly address this important question. They use an epidemiologic approach to assess the relationship between plasma ALA levels, dietary ALA consumption, and AF risk. In their study of 2899 subjects (65 years or older at study entry), there were 707 cases of incident AF during a 15 to 16 year (nearly 30 000 person‐years) follow‐up. No relationship was detected between plasma ALA and incident AF after correcting for age, sex, and a variety of clinical and demographic factors.

Strengths of the study include the size and age of the population, and the relatively large fraction of incident AF. Their results are consistent with an epidemiologic analysis suggesting that from age 40, there is approximately a 25% lifetime risk of developing AF for both men and women.^6^ Technical weaknesses of the study include the measurement of plasma ALA levels at only a single time point, and the reliance on food questionnaires to assess the level of dietary ALA consumption.

What are the implications of this study? The authors have concluded that neither ALA consumption nor plasma ALA levels are associated with AF risk.^5^ On the basis of their data, this seems to be a reasonable analysis and summary—for their study. However, we argue that the conclusion that ALA consumption is not related to AF risk is premature as a general statement. It is interesting to note that a similar epidemiologic study has recently reported a beneficial impact of dietary ALA with respect to risk of heart failure.^7^ AF and heart failure are frequently comorbid conditions. It seems likely that diets that substantially increase consumption of ALA while decreasing the intake of ω6 PUFA will have a beneficial impact on cardiovascular morbidity and mortality. However, optimal testing of this hypothesis would require careful longitudinal analysis of a cohort of patients with serial assessment of plasma PUFA levels, cardiovascular biomarkers, cardiac function, and electrophysiology.

Most enzymes that metabolize PUFAs do not distinguish between ω3 and ω6 isoforms. The rate of conversion is dependent on the relative abundance of each substrate. If the ω6 PUFAs are abundant, the conversion of short chain to long chain ω3 PUFAs will be inhibited. Short chain PUFA such as ALA (ω3) and linolenic acid (LA, ω6) are converted to long chain PUFA (eg., arachidonic acid [AA], EPA, DHA) by elongase and desaturase enzymes. The rate of conversion of ALA to EPA and DHA thus depends on the ratio of ω3:ω6 PUFA in the diet. The blood samples used in the ALA analysis of Fretts et al were collected in 1992–1993, a time at which the likelihood of finding an elderly cohort consuming the highest sources of ALA (chia and flax meal, or a diet high in greens and berries, which have a significant ALA content and a favorable ω3:ω6 ratio), was very unlikely. In their study, median ALA levels were only 0.1% (quartile 1) to 0.23% (quartile 4) of total plasma fatty acids. In contrast, LA constituted ≈20% of total plasma phospholipid fatty acids.

In a recent study evaluating the impact of a short‐term treatment with supplemental ω3‐PUFA on the incidence of AF following cardiac surgery,^8^ we found that the mean total plasma ω3:ω6 ratios were about 0.1. This means that there was on average ≈10 times more ω6‐PUFA in the plasma than ω3 PUFA. For ALA to have a meaningful impact on long chain ω3‐PUFA levels, the amount of ω6 PUFA that is consumed must be reduced and the amount of ALA consumed in the diet must be increased.

ALA is found in the chloroplasts of green leaves and in certain seeds.^9^ Although ALA is the most abundant fat on earth, the public is mostly unaware that they can consume large amounts of ALA—that add up—by eating plenty of greens, berries of all kinds, and especially the ALA‐rich foods like chia and flax meal. If they are unaware of the best sources of ALA, then it is unlikely they will consume enough to make a difference with respect to their risk of AF. Diets that reduce the intake of competing oils and increase the consumption of ALA‐rich plant products have been shown to significantly reduce the levels of biomarkers associated with risk of cardiovascular disease.^10–11^

What do these high ALA, low ω6 PUFA diets have in common? Might they offer a viable and sustainable strategy for reducing the risk of AF? Both the Ornish^7^ and Esselstyn^12–13^ diets are plant‐based, low‐fat, use no‐added oils, are high in ALA, and do not include nuts, a widely unrecognized high source of competing ω6 PUFAs. In effect, these diets eliminate the largest sources of LA and AA from the diet. While the Ornish/Esselstyn diets are widely recognized for their reduction in cardiovascular risk factors, they have been criticized for being unduly restrictive and/or unsustainable. The emphasis of these diets on patient education, careful instruction in diet principles and no‐oil cooking techniques, and efforts to provide group support makes them a viable and sustainable option for individuals who are motivated to make major changes to their diet and lifestyle.

2013 is a different world from the 1992–1993 world sampled in the Fretts' study.^5^ There is an active and growing grass roots, patient‐driven movement underway that recognizes the health benefits of a plant‐based diet, with an increasing number of authoritative physician‐and dietitian‐led websites, recipe sites, and social media support groups available to both educate and sustain the necessary dietary changes. Both authors of this editorial have experienced significant weight loss and seen personal improvements in cardiovascular biomarkers while following a plant‐based diet, and have found it easy to follow. One author (D.W.K.) maintains a nutrition/lifestyle blog and Facebook page. Comments from the many visitors to these sites defy the criticism of this type of diet as “restrictive and unsustainable.” Additional benefits of these diets include weight loss and improvements in blood pressure—both of which are significant risk factors for AF.

In summary, we conclude that, while the study of Fretts did not detect a relationship between ALA intake and risk of AF,^5^ systematic studies are needed to monitor the impact of diets that enhance ALA intake and promote its conversion to DHA. Only under these conditions can we rigorously determine whether dietary ALA has an impact on AF burden and AF progression. William Lands is a pioneer in the systematic study of ω3/ω6 physiology and biochemistry with more than 50 years experience in this field. In 1980, Dr. Lands and colleagues demonstrated that treatment with a diet containing high levels of EPA/DHA (from fish oil) decreased the incidence of ventricular arrhythmia following experimental myocardial infarction and reduced the size of the infarction.^14^ Dr. Lands is still very actively involved in studies that evaluate the health and socioeconomic impact of dietary fatty acid consumption choices.^15^ His advice is easily understood and clinically relevant: “if you understand that food energy causes transient inflammatory insults and that ω6s amplify that into chronic injury and ω3s moderate it, then you can tell people that the take home message is: Eat more ω3s; eat less ω6s; eat fewer calories per meal and stop smoking. That's it.” We concur with Dr. Lands, and suggest that adoption of a plant based diet rich in ALA may be one of the most sustainable and cost effective ways to accomplish this goal. Future studies will determine if such a diet has an impact on incident AF.
